# π phase shifter based on NbN-based ferromagnetic Josephson junction on a silicon substrate

**DOI:** 10.1038/s41598-020-70766-9

**Published:** 2020-08-13

**Authors:** Taro Yamashita, Sunmi Kim, Haruki Kato, Wei Qiu, Kouichi Semba, Akira Fujimaki, Hirotaka Terai

**Affiliations:** 1 Graduate School of Engineering, Nagoya University, Nagoya, Japan; 2 National Institute of Information and Communications Technology, Tokyo, Japan; 3 Advanced ICT Research Institute, National Institute of Information and Communications Technology, Kobe, Japan

**Keywords:** Electrical and electronic engineering, Superconducting devices, Quantum physics

## Abstract

In the field of superconducting electronics, a π phase shifter based on a ferromagnetic Josephson junction is expected to provide various advantages to classical and quantum superconducting devices. Here we report niobium nitride (NbN)-based ferromagnetic π junctions on a silicon (Si) substrate with a titanium nitride (TiN) buffer layer, which have applications to flux-bias-free flux quantum bits (qubits) and classical digital logic elements. We fabricated and characterized NbN/aluminum nitride (AlN)/NbN Josephson junctions, NbN/copper nickel (CuNi)/NbN ferromagnetic Josephson junctions, and superconducting quantum interference devices (SQUIDs) consisting of these junctions on the Si substrate. The fabricated NbN/AlN/NbN junctions showed a high junction quality suitable for qubit applications. Furthermore, the magnetic field dependence of the SQUID’s critical current indicated that the NbN/CuNi/NbN junction worked as a π phase shifter on the Si substrate.

## Introduction

Ferromagnetic Josephson junctions (superconductor/ferromagnet/superconductor junctions; SFS junctions) have been studied for many years from the perspective of fundamental physics of the interplay between superconductivity and magnetism^1–4^. A distinctive feature of SFS junctions is a “π state”, in which the superconducting phase difference is π in the ground state, contrary to conventional Josephson junctions with no phase difference. There have been many experimental demonstrations of the π state, including the temperature- or ferromagnetic barrier thickness-dependent cusp behavior of Josephson critical currents^5–10^, and a half-quantum-flux shift in the magnetic field modulation of the critical current in superconducting quantum interference device (SQUID) structures^11,12^. More recently, various device applications using SFS junctions in the π state (π junctions) have been proposed^13–19^. In superconducting logic circuits such as single-flux-quantum and adiabatic quantum-flux-parametron logic, further reductions of the consumption power^15^ and circuit area^16^ are expected by incorporating the π junctions into the circuits.

A notable application of the π junction is a π phase shifter for quantum bits (qubits)^14,17–19^. Among the various types of qubits, the flux qubit is a feasible candidate for realizing large-scale quantum computers with many qubits, due to its high anharmonicity and improved coherence time^20–22^. One of the biggest challenges is the necessity of an external half-flux-quantum bias to achieve qubit operation with the longest coherence time at the flux-insensitive point for each flux qubit. The π phase shifter provides a solution to this problem, and several theoretical proposals have been presented^17–19^. The common underlying idea is as follows. By inserting a π junction into the superconducting loop of the qubits, a spontaneous π phase shift is generated in the loop, which corresponds to a half-flux-quantum bias for conventional qubits; thus, qubits with the π junction operate at a flux-insensitive point without any external flux bias. This feature of the flux-bias-free flux qubit provides a great advantage for simultaneous operation of many qubits in large-scale quantum circuits.

Although the π junctions have been generally fabricated by using niobium (Nb) as superconducting electrodes^5–9^, we have demonstrated a niobium nitride (NbN)-based π junction on a magnesium oxide (MgO) substrate to realize flux-bias-free qubits^10^. Epitaxially grown NbN (100) films on a MgO (100) substrate have a smooth surface, unlike that of a polycrystalline Nb film, and thus these films achieve a clean superconductor/ferromagnet interface to provide improved controllability and reproducibility of the junction characteristics. Furthermore, NbN-based π junctions are compatible with superconducting qubits based on fully epitaxial NbN/aluminum nitride (AlN)/NbN (superconductor/insulator/superconductor; SIS) junctions grown on the MgO substrates^23,24^. Although a long coherence time is expected by adopting epitaxially grown Josephson junctions, it is known that the high-dielectric-loss MgO substrate is required to be replaced with a low-dielectric-loss material such as silicon (Si) or sapphire^25^. Regarding the SIS junctions, it has been reported that NbN/AlN/NbN junctions with a high quality comparable to those on the MgO substrates can be grown epitaxially on Si substrates with a titanium nitride (TiN) (200) buffer^26–28^. Thus, an important step for realizing flux-bias-free flux qubits with the π phase shifter is the development of a NbN-based π junction and high-quality SIS junctions on the same Si substrate.

In this paper, we demonstrated NbN-based π junction on the Si substrate by adopting the TiN buffer technique. We developed a hybrid process for fabricating SIS and SFS junctions on the same substrate. Based on this process, a “π-SQUID” structure, consisting of NbN/AlN/NbN junctions and a NbN/copper nickel (CuNi)/NbN junction as the π phase shifter, was successfully fabricated on the Si substrate. We measured the current–voltage characteristics of the junctions and the magnetic field dependence of the critical current for the π-SQUID. We observed a clear half-flux-quantum shift which was a direct evidence that the NbN/CuNi/NbN junction on the Si substrate worked as the π phase shifter.

### Fabrication and experimental setup

**Figure 1:**
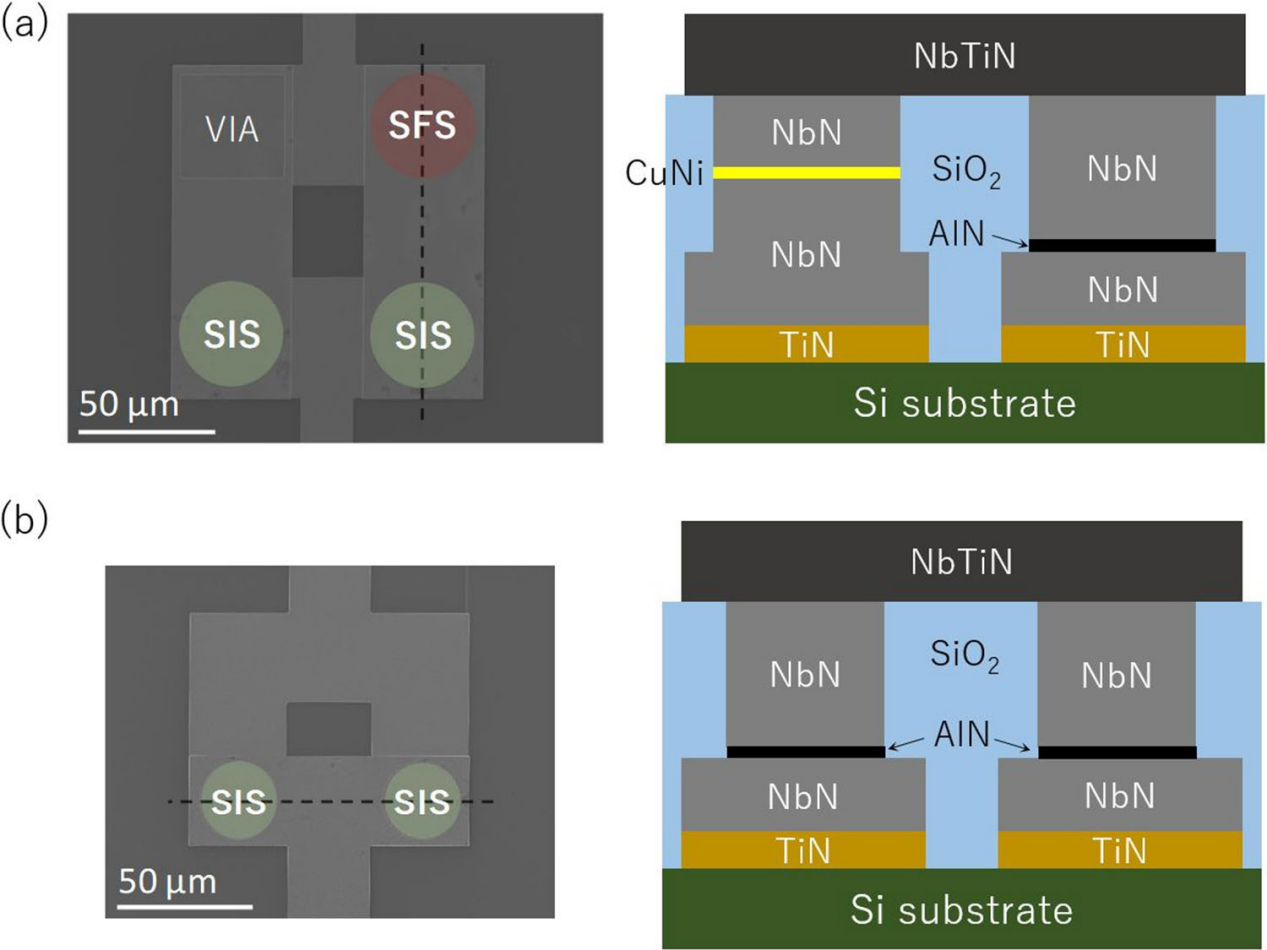
(**a**) False-color scanning electron microscopy (SEM) image (left) and cross-sectional diagram of the part indicated by the dashed line in the SEM image (right, not to scale) of the π-SQUID with two NbN/AlN/NbN (superconductor/insulator/superconductor, SIS) junctions and a NbN/CuNi/NbN (superconductor/ferromagnet/superconductor, SFS) junction. (**b**) SEM image (left) and cross-sectional diagram (right) of the dc-SQUID with two NbN/AlN/NbN junctions. Here the SiO_2_ layers indicated in (**a**) and (**b**) were removed by buffered hydrofluoric acid etching for the SEM observation.

In the present work, we used high-resistivity Si (100) substrates, which are commonly used for superconducting qubits. The substrate was treated with a mixed solution of sulfuric acid (H_2_SO_4_) and hydrogen peroxide (H_2_O_2_), followed by dipping in a diluted hydrofluoric (HF) solution to remove the native oxide and achieve hydrogen termination at the Si surface^26–28^. Within a few minutes, the substrate was installed in a load-lock chamber of a dc magnetron sputtering system with a background pressure less than 8 × 10^–8^ Pa. A TiN (200) film was deposited at a substrate temperature of 850℃ with a total pressure of 1 mTorr and an Ar:N_2_ gas flow ratio of 25:4. The deposited 50-nm-thick TiN film served as a buffer layer on the Si substrate^27,28^. Next, a NbN/AlN/NbN tri-layer was deposited on the TiN/Si substrate by using a process similar to that described in Ref^23^. The thicknesses of the base and counter NbN electrodes were 100 and 200 nm, respectively. The AlN thickness was ~ 1.8 nm for which the Josephson critical current density is expected to be around 10 A/cm^2^^23^. To define the SIS junctions, the tri-layer was patterned into a circular shape by an *i*-line stepper, and the counter NbN electrode was etched by reactive ion etching (RIE) using CF_4_ gas. Then, the base pattern of the SIS junction was made by RIE of the base NbN and TiN layers. Next, to fabricate the SFS junction in the superconducting loop with two Josephson junctions, a circular NbN/CuNi/NbN junction was made on top of the base pattern using a lift-off technique, as shown in Fig. 1a. The thicknesses of the base and counter NbN electrodes were 100 nm. The thickness of the CuNi barrier was set to 7.0 nm, to realize both the π state and a large critical current density more than several kA/cm^2^ enough to work as the π phase shifter for the conventional Josephson junctions (as described below)^10^. Then, a silicon dioxide (SiO_2_) film, which served as an insulating layer between the base and wiring layers, was deposited. We employed a planarization process with chemical mechanical polishing (CMP) to realize a reliable contact between the counter NbN electrode and the wiring layer. More details of the fabrication process of submicron junctions using the CMP process are described in Ref^28^. Finally, we deposited and patterned the wiring NbTiN layers.

In the fabricated samples, there were single SIS junctions with diameters of 1–40 μm, single SFS junction with a diameter of 4 μm, a direct-current SQUID (dc-SQUID, with two SIS junctions) shown in Fig. 1b,a π-SQUID (with two SIS junctions and one SFS junction) shown in Fig. 1a. The current–voltage characteristics of the junctions and magnetic field dependence of the critical currents of the dc-SQUID and π-SQUID were measured at 4.2 K in a magnetically shielded cryostat filled with liquid helium.

## Results and discussion

**Figure 2:**
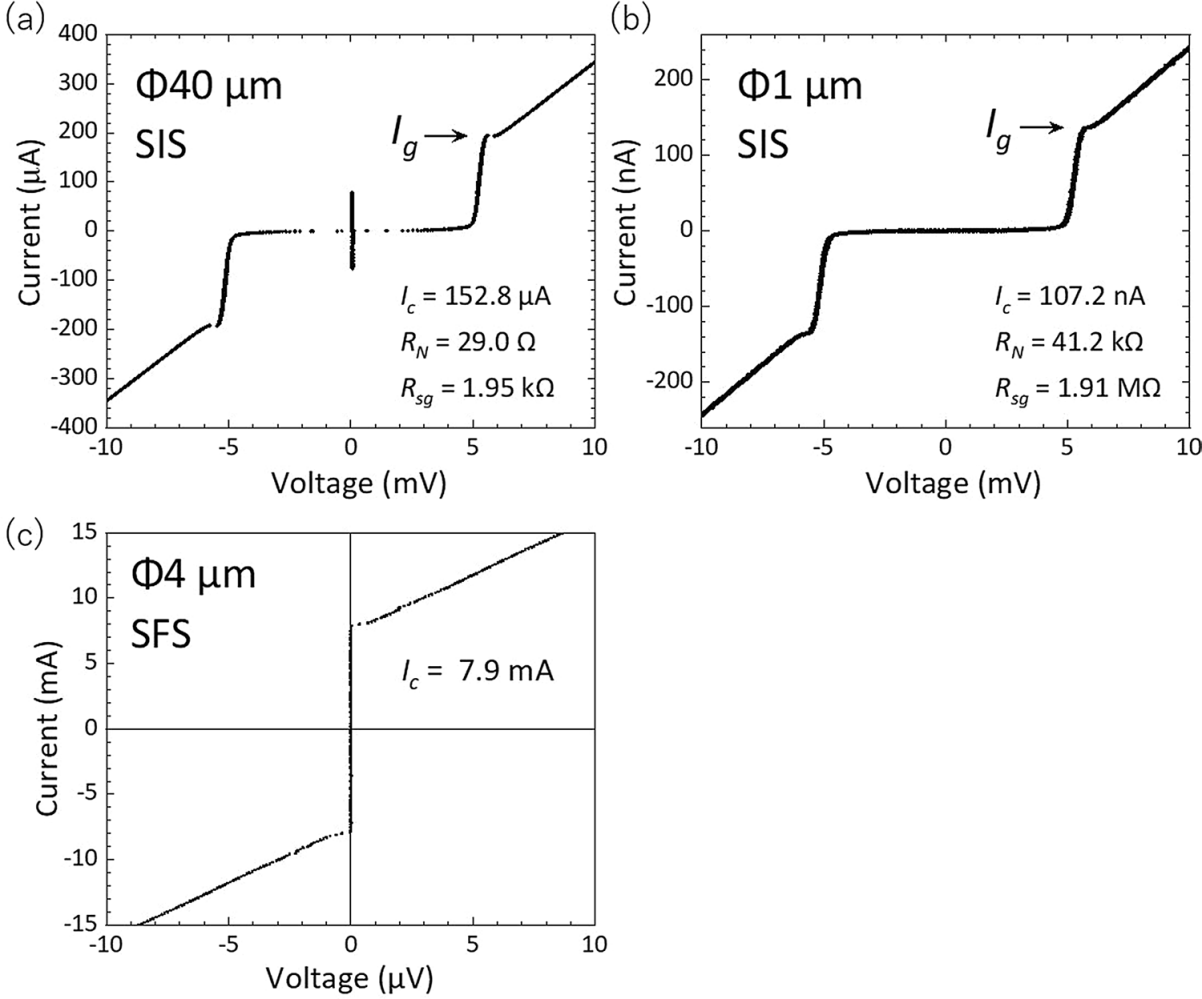
Current–voltage characteristics of NbN/AlN/NbN junctions with diameters of (**a**) 40 μm and (**b**) 1 μm at 4.2 K. (**c**) Current–voltage characteristics of a NbN/CuNi/NbN junction with a diameter of 4 μm.

Figure 2a,b show the current–voltage characteristics of the single NbN/AlN/NbN junctions. Because it is difficult to measure the true Josephson critical current ${I}_{c}$ at zero voltage due to the effects of noise and flux trapping, we estimated ${I}_{c}$ from $\left(\uppi /4\right){I}_{g}$, where ${I}_{g}$ is the current at the inflection point in the current–voltage characteristics (arrows in the figure)^23^. As shown in Fig. 2a, we obtained ${I}_{c}$ of 152.8 μA for the SIS junction with a diameter of 40 μm. This corresponds to the critical current density of 12.2 A/cm^2^, which is not far from the expected value of 10 A/cm^2^ for a 1.8-nm-thick AlN tunnel barrier. The gap voltage ${V}_{g}$, defined as the voltage at ${I}_{g}/2$, was 5.2 mV, and the quality factor ${R}_{sg}/{R}_{N}$ (where ${R}_{sg}$ and ${R}_{N}$ are the subgap resistance at 3 mV and the normal resistance at 10 mV, respectively) was 67.2. The obtained values indicate that the fabricated junctions were of similar quality to the high-quality NbN/AlN/NbN junctions on the MgO substrate, which have been used for the transmon qubits^23,24^. As shown in Fig. 2b, the small junction with a diameter of 1 μm showed a critical current density of 13.6 A/cm^2^, ${V}_{g}$ of 5.2 mV, and ${R}_{sg}/{R}_{N}$ of 46.4, similar to those of 40-μm-diameter junctions. This result indicates that the small Josephson junctions required for the qubits could maintain the high junction quality without degradation, even after several additional fabrication processes such as sputtering of the SFS film and planarization. We also measured the current–voltage characteristics of the single NbN/CuNi/NbN junction with a diameter of 4 μm. As shown in Fig. 2c, the critical current was 7.9 mA, and thus the critical current density was estimated to be 62.9 kA/cm^2^, which was much larger than that of SIS tunnel junctions, as expected for the 7.0-nm-thick CuNi metallic layer.

Next, we measured the magnetic-field modulation of the critical currents for the dc-SQUID and π-SQUID. To confirm that the SFS junction works as a π phase shifter from the modulations, we fabricated the dc-SQUID and π-SQUID with large (30- and 40-μm-diameter, respectively) SIS junctions, because it was difficult to measure the zero-voltage critical currents in SQUIDs with small SIS junctions.

**Figure 3:**
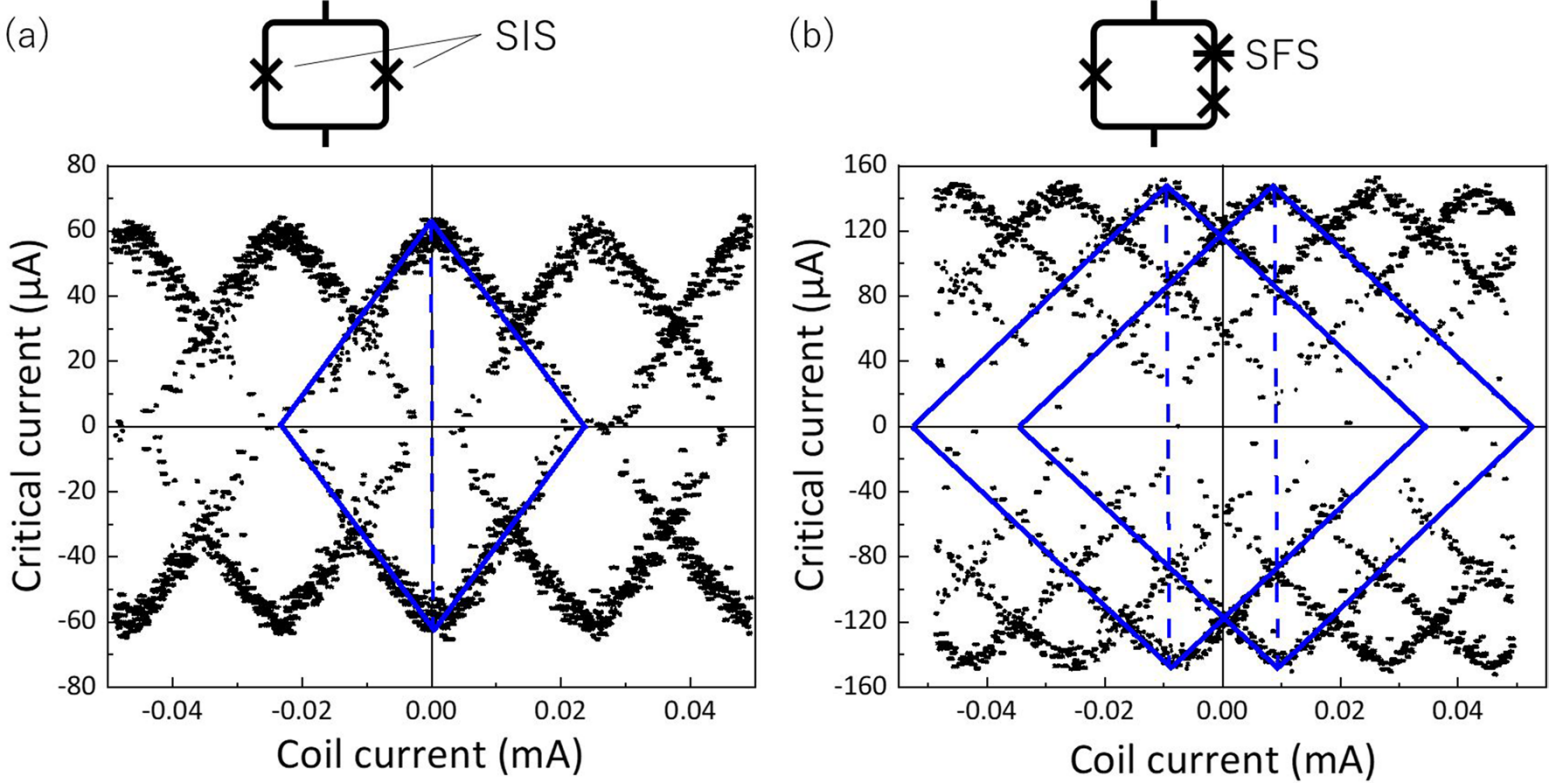
Magnetic field dependences of the critical current in (**a**) dc-SQUID and (**b**) π-SQUID at 4.2 K. The solid and dashed lines show the envelopes of the vortex states and the diagonal line of the envelopes, respectively.

Figure 3a shows the dependence of the positive and negative critical currents on the magnetic coil current for the dc-SQUID. In the modulation pattern for the dc-SQUID, the solid lines show the envelopes of the vortex states, and the intersection between the horizontal axis and the diagonal line (dashed line) of the envelope indicates the quantum number in the dc-SQUID. In the dc-SQUID, as expected, the intersections were located at coil currents corresponding to an integral multiple of the single flux quantum ${\Phi }_{0}$, i.e., ${n\Phi }_{0}$, where $n$ is an integer. In Fig. 3a, the critical currents did not drop to zero at half-flux-quanta. This is due to the relatively large inductance of the SQUID loop. By a brief estimation of the geometric and kinetic inductances for the dc-SQUID, we obtained around 40 pH as the total inductance. The geometric inductance is dominant because of a large inside hole size of around 20–30 μm in the SQUID loop as shown in Fig. 1b. The critical current of the SIS junctions in the dc-SQUID is 30 μA, so the product of the inductance and the critical current becomes around ${0.6\Phi }_{0}$. This is consistent with the result in Fig. 3a because the critical current at half-flux-quanta becomes half of the maximum value theoretically when the product is ${0.5\Phi }_{0}$.

In the π-SQUID, when the Josephson energy (i.e., the critical current) of the SFS π-junction is much larger than those of the other SIS junctions, the phase difference at the SFS junction retains its value of nearly π^19^. The fabricated sample satisfied this condition because the critical current density of the SFS junction was more than three orders of magnitude higher than that of the SIS tunnel junctions. As a result, a spontaneous π phase shift appears in the total phase of the superconducting loop; thus, a half-flux-quantum (${\Phi }_{0}/2$) shift is expected in the modulation pattern of the critical current, relative to that of the conventional dc-SQUIDs. As shown in Fig. 3b, in the magnetic modulation pattern for the π-SQUID, the intersections between the horizontal axis and the diagonal lines (dashed lines) were equally shifted in positive and negative coil-current directions from the origin by a half period of the modulation pattern. This shift corresponds to the half flux quantum $\pm {\Phi }_{0}/2$. The obtained result provides direct evidence that the inserted NbN/CuNi/NbN junction was actually in the π state and worked as the π phase shifter in the π-SQUID.

Although the current noises shown in Figs. 3a,b were relatively larger than those in Fig. 2a,b, this would be mainly due to the imperfect filtering in the measurement setup, not intrinsic one. The measurements for the modulation pattern have been performed by using the cryogenic probe which had no resistors to filter the noises in the signal lines between 4.2 K and the room temperature whereas the current–voltage characteristics shown in Fig. 2a,b have been measured by the probe with 1-kΩ resistors at 4.2-K sample holder.

We made the relatively large (40-μm-diameter) SFS junction to demonstrate the π phase shifter for sure in the present work. In the actual flux qubits, the critical currents of the SIS junctions and circulating currents in the qubit loop are as small as around 100 nA^21^. To work as the passive π phase shifter in the qubit, the SFS junction is required to have the critical current much larger than these values. The critical current density of the fabricated SFS junction was 62.9 kA/cm^2^, so even in much smaller SFS junctions, e.g., diameters of around 1 μm, the critical current is three orders of magnitude higher than that of SIS junction, which satisfies the requirement to act as a π phase shifter. Because the typical size of the SIS junction in the qubits is several hundred nanometers, the SFS junction with the comparable size is small enough not to prevent the qubit integration. Furthermore, sub-μm-diameter SFS junctions will not suffer from the stray flux from the CuNi barrier because the magnetic domain size of the CuNi film is around 100 nm^14,29^.

## Conclusion

In conclusion, we demonstrated a NbN-based π junction on a Si substrate with a TiN buffer. Using our hybrid fabrication process for SIS and SFS junctions on the same substrate, we fabricated a NbN/AlN/NbN (SIS) junction, a NbN/CuNi/NbN (SFS) junction, and SQUIDs with/without the SFS junction on the Si substrates. The developed SIS junctions showed high junction qualities, similar to those of the junctions on the MgO substrate. From the magnetic field dependences of the critical current for the SQUID with the SFS junction, it has been demonstrated that the NbN/CuNi/NbN junction surely worked as the π phase shifter on the Si substrate. The results shown in the present work provide physical and technical bases for realizing a large-scale superconducting quantum circuit with a number of flux-bias-free flux qubits.
